# In Sickness and in Health: Perineuronal Nets and Synaptic Plasticity in Psychiatric Disorders

**DOI:** 10.1155/2016/9847696

**Published:** 2015-12-29

**Authors:** Harry Pantazopoulos, Sabina Berretta

**Affiliations:** ^1^Translational Neuroscience Laboratory, Mclean Hospital, Belmont, MA 02478, USA; ^2^Department of Psychiatry, Harvard Medical School, Boston, MA 02215, USA; ^3^Program in Neuroscience, Harvard Medical School, Boston, MA 02215, USA

## Abstract

Rapidly emerging evidence implicates perineuronal nets (PNNs) and extracellular matrix (ECM) molecules that compose or interact with PNNs, in the pathophysiology of several psychiatric disorders. Studies on schizophrenia, autism spectrum disorders, mood disorders, Alzheimer's disease, and epilepsy point to the involvement of ECM molecules such as chondroitin sulfate proteoglycans, Reelin, and matrix metalloproteases, as well as their cell surface receptors. In many of these disorders, PNN abnormalities have also been reported. In the context of the “quadripartite” synapse concept, that is, the functional unit composed of the pre- and postsynaptic terminals, glial processes, and ECM, and of the role that PNNs and ECM molecules play in regulating synaptic functions and plasticity, these findings resonate with one of the most well-replicated aspects of the pathology of psychiatric disorders, that is, synaptic abnormalities. Here we review the evidence for PNN/ECM-related pathology in these disorders, with particular emphasis on schizophrenia, and discuss the hypothesis that such pathology may significantly contribute to synaptic dysfunction.

## 1. Introduction

The classic view of psychiatric disorders as “neuronal” disorders has been challenged in recent years by rapidly emerging evidence pointing to the involvement of the extracellular matrix (ECM), glial cells, and their interactions [1–8]. This evidence represents a significant departure from mainstream views and is driving the field toward a growing understanding of these elements as closely interacting components of functional units, such as the “tetrapartite synapse.” This latter term, originally proposed by Dityatev et al. [9], aptly describes the functional unit composed of the of pre- and postsynaptic terminals, astroglial processes, and synaptic/perisynaptic ECM complexes [10–14]. Here, we review evidence for the involvement of the ECM in psychiatric disorders and focus on the hypothesis that ECM abnormalities may contribute to a critical pathological component shared by a large subgroup of these disorders, that is, disruption of synaptic functions [15–21]. First, evidence for ECM abnormalities in schizophrenia, the main focus of these authors' studies, is discussed, with particular emphasis on loss of perineuronal nets (PNNs) in several brain regions in this disorder. We then briefly review evidence for a significant involvement of synaptic pathology in this disorder and follow with a discussion on the potential mechanisms linking such pathology to ECM/PNN abnormalities. Finally, evidence for ECM involvement in other psychiatric disorders is reviewed, with reference to molecular families known to play a role in synaptic functions. The specific patterns and causes of ECM abnormalities in each of these disorders are not yet well understood and may be disorder-specific. We postulate that overlapping patterns of ECM/PNN abnormalities may underlie shared synaptic pathology in these disorders.

It should be emphasized here that synaptic regulation is only one of several critical functions performed by the ECM during pre- and postnatal brain development as well as adulthood (for reviews see [22–30]). Thus, in addition to synaptic dysregulation, the consequences of brain ECM abnormalities may be complex and far-reaching, spanning from disruption of axonal guidance, neuronal differentiation, and migration in early brain development to circuit consolidation and closure of critical periods in postnatal development and finally axonal signal conduction and regulation of the blood/brain barrier in the adult brain [1, 2, 31–38].

## 2. Schizophrenia

### 2.1. ECM/PNN Abnormalities in Schizophrenia

Schizophrenia is a chronic, severe, and disabling brain disorder characterized by psychotic symptoms and disruptions of normal emotions and behaviors. Growing evidence points to ECM abnormalities as a component of the core pathophysiology of schizophrenia. Converging results from human genetic and postmortem studies show genetic vulnerabilities for genes encoding several key ECM molecules, including chondroitin sulfate proteoglycans (CSPGs), Reelin, semaphorin 3A, integrins, and remodeling enzymes, as well as dysregulated expression of these molecules in glial cells, and disruption of organized ECM structures such as PNNs (see references below). Animal models indicate that these abnormalities may have far-reaching consequences on neural circuits involved in schizophrenia [39–41]. These findings are briefly reviewed below.

#### 2.1.1. CSPGs

**Figure 1:**
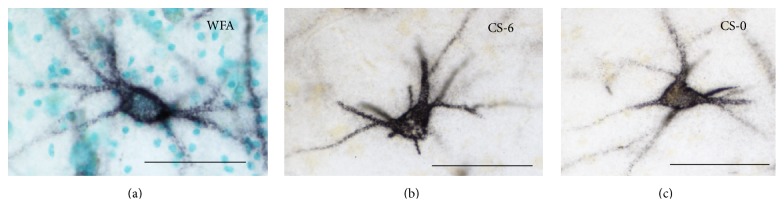
PNNs in the human amygdala. (a) PNN labeled with* wisteria floribunda* agglutinin (WFA) lectin, which binds to N-acetyl-galactosamine on the terminal end of chondroitin sulfate chains. (b) PNN immunolabeled using the mAB antibody 3B3 against native chondroitin sulfate motifs specific for chondroitin-6-sulfate (CS-6). 3B3-immunolabeled PNNs are more numerous and show a much broader distribution in the human amygdala with respect to those labeled with WFA. (c) PNN immunolabeled with mAB antibody 1B5, raised against nonsulfated chondroitin sulfate (CS-0) (3B3 and 1B5 are a generous gift from Dr. Bruce Caterson, University of Cardiff, UK). Scale bar = 50 *μ*m.

**Figure 2:**
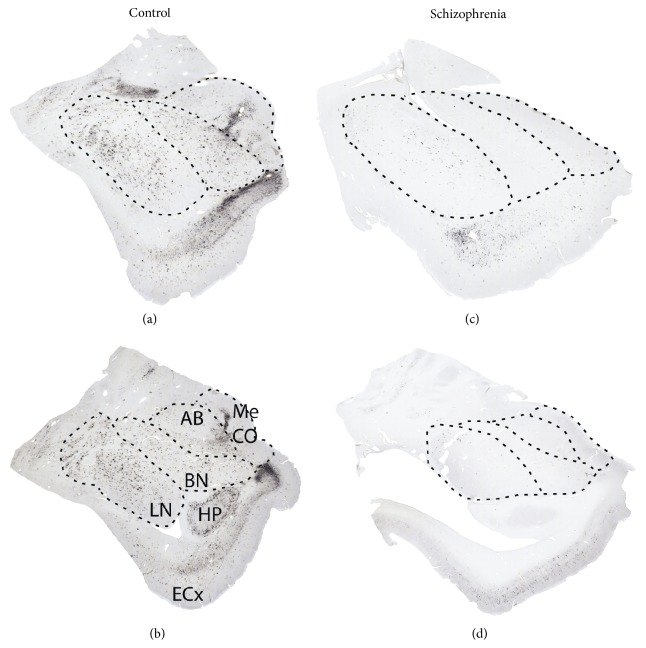
Decreased CS-6 in the amygdala of subjects with schizophrenia. In the amygdala of control subjects, CS-6(3B3)-labeled PNNs and glial cell clusters are distributed in the lateral (LN), basal (BN), accessory basal (AB), and corticomedial (CO-Me) nuclei (a, b). In subjects with schizophrenia, marked decreases of PNNs and glia cell clusters immunolabeled for CS-6(3B3) were observed in the amygdala of (c, d) [55]. ECx: entorhinal cortex. HP: hippocampus.

In subjects with schizophrenia, we first reported marked decreases of CSPG-labeled PNNs in the amygdala and entorhinal cortex [42], interconnected brain regions involved in emotion-related learning and associative sensory information processing and in the pathophysiology of schizophrenia [43–47]. In this study, PNNs were detected using the lectin* wisteria floribunda* agglutinin (WFA; Figure 1), which labels PNNs predominantly associated with GABAergic neurons expressing the calcium binding protein parvalbumin (PVB) [48–54]. Consistently, lower numbers of WFA-labeled PNNs were observed in the lateral nucleus of the amygdala and the superficial layers of the entorhinal cortex, where these interneurons are primarily located [42]. A similar distribution pattern of PNN decreases was detected using antibodies against aggrecan, one of the main CSPGs in the brain [55]. In contrast, immunolabeling with antibodies raised against a specific chondroitin sulfate 6 (CS-6; Figure 1) pattern revealed more extensive PNN distribution in the normal human amygdala and decreases in subjects with schizophrenia, including not only the lateral nucleus but also the basal, accessory basal, cortical, and medial amygdala nuclei [55] (Figure 2). PNN reduction in this latter nucleus is of particular interest, as it suggests that GABAergic projection neurons are also affected by PNN abnormalities. Notably, PNN decreases were not accompanied by neuron number reductions [42, 43], pointing to actual loss, or altered neurochemical composition, of PNNs. Lower densities of WFA-labeled PNNs were also detected in layers III and V of the prefrontal cortex of subjects with schizophrenia [56]. Interestingly, the visual cortex did not show similar changes [56], suggesting that while being widespread PNN decreases may spare brain regions that are not heavily involved in the pathology of schizophrenia.

In addition to CSPG-labeled PNN decreases, markedly altered CSPG expression in schizophrenia was also detected in glial cells and “glial clusters” in the amygdala, as well as in olfactory receptor neurons in the olfactory epithelium [42, 55, 57]. In parallel to PNN-related findings, these changes did not appear to depend on altered cell numbers and did not depend on disease-related confounding factors such as exposure to pharmacological treatment, substance abuse, onset and duration of the illness, and so forth, further supporting the idea that altered CSPG expression in schizophrenia may represent a core feature of this disorder [42, 55, 57].

Further support for CSPG involvement in schizophrenia comes from molecular dissection of the neuregulin-ErbB4 pathway, which revealed an association with a genetic polymorphism in PTPRZ1, the gene encoding for receptor phosphotyrosine phosphatase beta/zeta (RPTPbeta) with schizophrenia [58]. RPTPbeta is a transmembrane CSPG shown to play a role in synaptic plasticity and learning [59–63]. Increased mRNA expression of PTPRZ1 has been reported in the amygdala and prefrontal cortex of subjects with schizophrenia [39, 42]. Genetic studies have further identified associations of schizophrenia with the genes encoding for the CSPGs neurocan (NCAN) and neuroglycan-C [64, 65], suggesting that abnormal CSPG expression in schizophrenia may be due, at least in part, to genetic factors.

#### 2.1.2. Reelin

The glycoprotein Reelin is arguably one of the ECM molecules most extensively investigated in schizophrenia and other psychiatric disorders. In subjects with schizophrenia, it has been widely reported to be decreased in a number of cortical areas [66–70]. Several studies have shown that Reelin expression is reduced concurrently with GAD67, one of the main synthetic enzymes for GABA, in cortical GABAergic interneurons, and that these changes may be the consequence of an epigenetic hypermethylation of RELN and GAD67 promoters in these interneurons [70–74]. Notably, Reelin expression was found to be decreased in interstitial white matter neurons, in a study that also confirmed increased density of these neurons in schizophrenia [75, 76]. Together, these studies elegantly link a disruption of Reelin expression in GABAergic neurons to dendritic spine loss and altered neuronal migration in schizophrenia.

#### 2.1.3. Semaphorins

Members of the semaphorin family, and semaphorin 3a in particular, have also been shown to be altered in schizophrenia, potentially in conjunction with Reelin [66, 77]. In particular, increased semaphorin 3A and decreased Reelin expression were detected in the cerebellum of subjects with schizophrenia, while altered expression of multiple members of the semaphorin family was observed in the prefrontal cortex [66]. Genetic polymorphisms of genes encoding for semaphorin 3D and semaphorin receptor plexin A2 have also been associated with schizophrenia ([78, 79], but see also [80]).

#### 2.1.4. Integrins

Integrins, a family of heterodimeric cell adhesion molecules (CAMs) consisting of several different *α*- and *β*-subunits, interact with ECM molecules to carry out a multitude of developmental and adult brain functions. Support for the idea that integrins may be implicated in the pathology of schizophrenia comes in part from genetic studies pointing to association of this disorder with a number of integrin gene variants, such as SNPs in the ITGA8 and ITGB3 genes [81, 82]. Additional evidence supporting the involvement of integrins in schizophrenia includes increased expression of integrin *α*(IIb) and *β*(IIIa) in first episode subjects with schizophrenia and, notably, abnormal cell adhesion in cultures from olfactory mucosa biopsies from patients with this disorder, which was ameliorated by antibodies blocking integrins [83, 84].

#### 2.1.5. Matrix Metalloproteases

Proteolytic ECM remodeling, shown to play a key role in synaptic plasticity, is mediated by matrix metalloproteases (MMPs), “a disintegrin and metalloproteases” (ADAMs), and “a disintegrin and metalloproteases with a thrombospondin motif” (ADAMTS), through their substrates, such as CAMs, CSPGs, and ECM receptors [85–89]. Several of these enzymes have been implicated in the pathophysiology of schizophrenia. Elevated levels of MMP-9, and “tissue inhibitor of metalloproteinases 1” (TIMP-1) which blocks MMP-9 activity, were reported in blood samples from subjects with this disorder [90, 91]. Increased MMP-9 blood serum levels were also identified in treatment resistant patients [92]. A recent gene expression profiling study of the superior temporal gyrus showed altered mRNA expression of MMPs and ADAMTSs in schizophrenia, including MMP-16 [93]. The possibility that genetic vulnerability may contribute to altered expression of matrix metalloproteases in schizophrenia is supported by converging results from several recent genetic association and genome-wide association studies showing that gene variants encoding for a number of these enzymes, including ADAMTSL3, ADAMTS12, ADAMTS16, ADAM22, and MMP-16, may be associated with this disorder [94–98].

#### 2.1.6. Evidence for ECM Abnormalities from Animal Model Studies

Consistent with human studies, animal models provide evidence that abnormalities affecting the ECM, and CSPGs in particular, may contribute to the pathophysiology of schizophrenia. Transgenic mice overexpressing PTPRZ1 show numerous anatomical and behavioral abnormalities also observed in this disorder, including delayed oligodendrocyte maturation, working memory deficits, and altered glutamatergic, dopaminergic, and GABAergic activity [39]. Experimentally induced enzymatic PNN digestion in the mouse hippocampus mimics several functional abnormalities, including increased activity of dopamine neurons in the ventral tegmental area, which is suspected to occur in schizophrenia [40]. Finally, a rodent model for oxidative stress in schizophrenia showed that PNNs protect neurons expressing PVB from oxidative stress, while at the same time they are vulnerable to it [41]. Thus, loss of PNNs may render these neurons more susceptible to the excitotoxic effects of oxidative stress believed to occur in schizophrenia [99–101].

### 2.2. Synaptic Pathology in Schizophrenia

Solid and growing evidence shows that disruption of synaptic functions represents a core component of the pathology of schizophrenia. Altered synaptic transmission of key CNS neurotransmitters, including glutamate and GABA, altered expression of synaptic molecules, and loss of dendritic spines have been consistently observed in schizophrenia [16, 102–111]. These interlinked components are briefly reviewed below and placed in the context of ECM/PNN abnormalities.

#### 2.2.1. Glutamatergic Synaptic Signaling and GABAergic Inhibitory Neurons

Several neurotransmitter systems have been implicated in the pathophysiology of schizophrenia. For the purpose of this review, we focus on the involvement of glutamatergic transmission and GABAergic inhibitory neurons in schizophrenia and discuss the potential contribution of ECM/PNN pathology to abnormalities affecting these systems. Importantly, abnormalities affecting these neurotransmitters are closely linked to one another. For instance, GABAergic interneurons powerfully regulate intrinsic information processing and glutamatergic efferents (e.g., [112–115]). Conversely, these interneurons are particularly sensitive to glutamate NMDA receptor hypofunction [116, 117].

Abnormalities affecting the GABAergic system have been consistently reported in several brain regions in schizophrenia [115, 118–129]. These include decreases of inhibitory neurons, loss of GABAergic terminals, and expression of glutamatergic receptors on distinct populations of GABAergic interneurons [115, 130, 131]. The regulation of glutamatergic inputs to these neurons, and PVB-positive neurons in particular, has been a specific focus of attention in schizophrenia, as these inputs play a key role in controlling synchronous oscillations at gamma band frequencies, known to be affected in schizophrenia, and are critical to information processing in cortical circuits involved in this disorder [122, 126, 132, 133].

Altered expression of NMDA and, perhaps to a lesser extent, AMPA receptor proteins has been reported in subjects with schizophrenia, pointing to a disruption of synaptic glutamate signaling networks in this disorder [134–142]. In addition, and perhaps more consistently, the expression of a large number of glutamate receptor accessory proteins, including proteins associated with the postsynaptic density, is altered in several brain regions in people with schizophrenia (for review see [141]). Converging evidence from postmortem, genetic, and animal models also points to abnormal expression of the NMDA receptor coagonists, D-serine and glycine, and the endogenous glycine modulatory site antagonist kynurenic acid [143–150]. This well-replicated finding resonates with intriguing evidence for ECM-mediated modulation of the NMDA receptor glycine site [151].

### 2.3. Potential Contribution of ECM Abnormalities to Synaptic Glutamatergic Transmission on GABAergic Neurons

**Figure 3:**
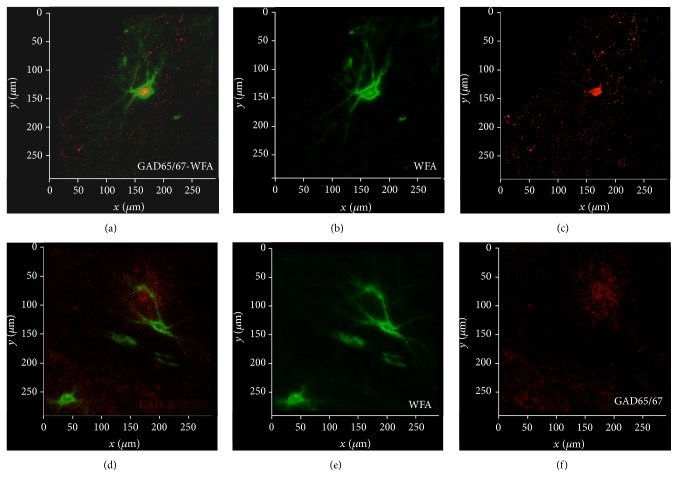
PNNs are associated with heterogeneous neuronal populations. Confocal micrographs depicting WFA-labeled PNNs surrounding GABAergic neurons expressing GAD65/67 in the healthy human amygdala (a–c). Consistently, WFA-labeled PNNs have been shown to be typically associated with neurons expressing parvalbumin. However, we have previously reported that a small subpopulation of these PNNs ensheathes parvalbumin-negative neurons [152]. Preliminary findings shown in (d)–(f) suggest that these neurons do not express GAD65/67 and may therefore correspond to excitatory neurons.

PNNs tightly surround synaptic contacts on the somata, dendrites, and proximal axon segment of distinct populations of neurons. These consist of several GABAergic interneuron populations, including, but not limited to, those expressing PVB or somatostatin, and GABAergic projection neurons such as those in the reticular nucleus of the thalamus, the central nucleus of the amygdala, and Purkinje cells in the cerebellum [2, 48, 153–158]. A subset of excitatory corticocortical pyramidal cells and spinal cord motor neurons have also been found to bear PNNs [159]. It is likely that the range of neuronal populations associated with PNNs has not yet been fully accounted for, particularly as the molecular and structural heterogeneity of these ECM structures is still not well understood. For instance, in the human amygdala, a small population of WFA-labeled PNNs ensheathes neurons lacking expression of glutamic acid decarboxylase (GAD) and thus likely represented excitatory neurons (unpublished results; Figure 3). In addition, we recently reported that PNNs labeled with an antibody (3B3) targeting a native chondroitin-6-sulfate motif are far more numerous and widely distributed than those labeled with the lectin* wisteria floribunda* agglutinin (WFA) and well represented in amygdalar nuclei virtually devoid of PVB-positive neurons [55]. Therefore, abnormalities affecting PNNs may potentially impact a broad variety of inhibitory, as well as excitatory, neuronal populations. Converging evidence suggests that, in schizophrenia, ECM/PNN components known to regulate glutamatergic and GABAergic inputs on GABAergic neurons are abnormal, potentially contributing to intrinsic information processing and activity outflow.

### 2.4. Regulation of Glutamatergic and GABA Synapses by PNNs and Perisynaptic ECM Condensations

Growing evidence points to a key role played by PNNs in synaptic regulation, particularly of glutamatergic synapses (for extensive reviews see [11, 12, 38, 160, 161]). In particular, ECM/PNNs affect the diffusion of glutamate receptors laterally within the plasma membrane, as well as receptor clustering within the synapse, thus controlling a fundamental mechanism of synaptic regulation and plasticity [162, 163]. It is thought that this key PNN function may be accomplished by a combination of passive and active mechanisms [160, 162]. Although less is currently known with regard to PNN regulation of GABAergic inputs, a recent study reported that PNN enzymatic digestion increases the number of inhibitory synapses on PVB-positive interneurons [164]. We briefly describe below some examples according to their potential relevance to schizophrenia. We suggest that, in schizophrenia, concurrent disruption of CSPG expression and molecular families interacting with PNN components may contribute synergistically to glutamatergic synapse dysregulation on neurons associated with PNNs.

#### 2.4.1. PNNs as a Passive Barrier

Highly viscous CSPGs, and other ECM/PNN components, form an effective passive diffusion barrier, controlling the lateral diffusion exchanges of AMPA receptors between the synaptic and extrasynaptic compartments [162]. By restricting the lateral diffusion of AMPA receptors from the extrasynaptic space to the synapse, PNNs allow synaptic desensitization during high frequency firing [160, 162, 165]. Consistent with this function, PNN enzymatic digestion results in increased excitability of interneurons [166]. In pathological states, such as schizophrenia, PNN disruption may result in unregulated lateral membrane diffusion of AMPA receptors, thus impacting excitatory synaptic activation and resembling a more “juvenile” state of synaptic regulation.

#### 2.4.2. Chondroitin Sulfate Proteoglycans

CSPGs are key contributors to the composition of PNNs [167–169] and are also found in other structures described as perisynaptic coats [154, 170–174]. An increasing number of studies show that these molecules are critically involved in the regulation of synaptic plasticity. For example, electrophysiological recordings from in vitro mouse hippocampal slices treated with chABC to remove CSPGs show a twofold decrease in long term potentiation (LTP) but not in short term plasticity [175]. A similar decrease of LTP was also observed in mice lacking a key PNN component, tenascin-R, suggesting that CSPG regulation of long term synaptic plasticity occurs through modulation of PNN composition [175].

Studies focused on specific CSPG molecules and CS-sulfation reveal a complex role in developmental and adult regulation of synaptic plasticity. For example, overexpression of CS-6 sulfation in mice leads to failure to instate an adult form of restricted plasticity, resulting in abnormally persistent synaptic plasticity and reduced PNN formation [176]. In the cortex and hippocampus, expression of the CSPG PTPRZ1 was found to be associated with synaptic remodeling [59–62]. Knockout of PTPRZ1 in mice results in enhanced LTP and deficits in spatial learning exclusively in adults [63]. In contrast, mice overexpressing PTPRZ1 show hippocampal LTP deficits, as well as a number of molecular, anatomical, and behavioral abnormalities reminiscent of those observed in subjects with schizophrenia [39]. Together with the increased expression of PTPRZ1 in subjects with this disorder [39, 42], these findings are consistent with the possibility that increased PTPRZ1 expression may contribute to deficits in synaptic plasticity in schizophrenia. Other CSPGs have also been shown to regulate plasticity. Adult mice deficient for the CSPG brevican display deficits in hippocampal early stage LTP and decreased PNN formation [177], whereas mice deficient for neurocan display decreased late stage LTP but normal PNN formation [178]. Further evidence comes from studies showing that brevican and versican are increased in the hippocampus of rats during memory retrieval in the Morris water maze spatial memory task [179]. Overall, altered expression of CSPGs and CS-sulfation patterns have the potential to contribute to disregulated synaptic plasticity in psychiatric disorders during developmental stages, particularly during critical periods of plasticity, as well as adult regulation of synaptic plasticity.

#### 2.4.3. Reelin

Perhaps one of the most well-replicated findings in the pathophysiology of schizophrenia and, incidentally, autism is a disruption of Reelin expression [66–74, 76]. In the adult brain, Reelin is secreted in the ECM by subpopulations of GABAergic interneurons and takes part in the composition of at least a subpopulation of PNNs [180–182]. Reelin's effects are mediated through its main lipoprotein receptors, apolipoprotein E receptor 2 (ApoER2) and very-low-density lipoprotein receptor (VLDLR) [183, 184], as well as through the CAMs of the integrin family and the Src family kinases [184–188]. Accumulating evidence shows that secreted Reelin powerfully enhances LTP and glutamatergic synaptic transmission by regulating NMDA and AMPA receptors [187, 188]. For instance, Reelin regulates the composition of NMDA receptors, controlling the predominance and/or phosphorylation of the NR2 NMDA receptor subunits, and enhances AMPA responses by increasing the number of AMPA receptors on the postsynaptic membrane [180, 188].

#### 2.4.4. Integrins

Interactions between integrins and ECM/PNN components, including Reelin, thrombospondins, fibrinogen, and others, have been shown to regulate synaptic glutamatergic transmission [160]. Notably, integrin signaling is bidirectional; that is, it can activate intracellular signaling pathways in response to changes in the extracellular environment and impact on cell adhesion in response to intracellular signaling [189]. This may allow integrins to play complex roles in synaptic plasticity, including carrying out structural and functional changes that accompany LTP [189–191]. Integrin-ECM interactions have been shown to regulate AMPA receptor internalization, surface mobility of NMDA receptor subunits, and synaptic dwell time of glycine receptors and their scaffolding molecule gephyrin [151, 192, 193].

#### 2.4.5. Neuronal Activity-Regulated Pentraxin

An intriguing example of interactions between ECM molecules and factors regulating synaptic plasticity is represented by the “neuronal activity-regulated pentraxin” (NARP). NARP is an immediate early gene in which its protein product is secreted in an activity-dependent manner by excitatory terminals onto GABAergic PVB-positive neurons, where it promotes clustering of AMPA receptor subunits [194, 195]. This mechanism regulates homeostatic scaling of excitatory inputs so that increased network activity strengthens the excitatory inputs on PVB-positive interneurons, in turn powerfully inhibiting excitatory projection neurons [195]. PNNs ensheathing PVB-positive neurons are critical to the maintenance of high levels of NARP at excitatory synapses on these neurons [195]. CSPG enzymatic digestion markedly decreased NARP at excitatory synapses on PVB-positive neurons without decreasing the overall NARP expression [195]. In subjects with schizophrenia, marked Narp mRNA decreases were reported in the prefrontal cortex [196], a brain region where PNN decreases were also detected [56]. Although a causal relationship between these two findings has not been established thus far, it is reasonable to postulate that, in conjunction, NARP and PNN decreases may synergistically impact glutamatergic synapses on PVB-positive neurons in schizophrenia.

#### 2.4.6. Matrix Metalloproteases

Secreted extracellular matrix proteases, such as “tissue plasminogen activator” (tPA) and MMPs, affect excitatory transmission. For instance, tPA has been found to play a role in LTP through several mechanisms, including cleavage of the NR1 subunit of the NMDA receptor, resulting in potentiation of NMDA current, and cleavage of proBDNF resulting in availability of mature BDNF [197–200]. Altered levels of tPA have been reported in subjects with schizophrenia [201–203]. Although it is not currently known whether these abnormalities are linked to comorbidities, such as alcoholism, inflammatory, and autoimmune disorders, or metabolic disorders [201–203], these findings support the intriguing possibility that tPA abnormalities may contribute to a disruption of glutamatergic transmission in schizophrenia. Interestingly, the amygdala, where marked PNN decreases were detected in schizophrenia, is particularly enriched in tPA [42, 55, 204–206]. MMP-9 has also been shown to powerfully regulate synaptic plasticity and LTP in particular, a role mediated by 1-containing integrin receptors [207]. Interestingly, MMP-9 is transiently released in response to enhanced neuronal activity and impacts both synaptic potentiation and dendritic spine enlargement in a dependent manner [207, 208]. As discussed above, the possibility that MMP-9, as well as other MMPs with potentially related functions, may represent genetic vulnerabilities in schizophrenia has been gaining evidence in recent times [98, 208–210].

### 2.5. Loss of Dendritic Spines in Schizophrenia

Marked reductions of dendritic spines have been consistently reported in schizophrenia, encompassing several cortical areas, including prefrontal and auditory cortical areas and the hippocampus [104, 109–111, 211]. In addition, the expression of postsynaptic density (PSD) proteins, such as PSD95 and Homer-1, and associated glutamate signaling pathway proteins has been shown to be altered in subjects with this disorder, as well as with autism spectrum disorders [107, 108]. In support of the idea that these changes reflect a structural loss of dendritic spines, altered expression of molecules involved in the actin cytoskeleton has been reported in subjects with schizophrenia [102, 103, 105].

### 2.6. Potential Contribution of ECM Abnormalities to Loss of Dendritic Spines

Dendritic spines contain the membrane-associated postsynaptic density (PSD) and its associated network of neurotransmitter receptors and downstream signaling molecules and are supported by a mesh of filamentous F-actin and scaffolding proteins [212–218]. ECM proteins, their surface receptors, and remodeling ECM enzymes play a critical role in regulating dendritic spine plasticity in adulthood (see review by [197]). CAMs, among which integrins are perhaps the most well studied, link the PSD to the actin cytoskeleton on one side and to the ECM and presynaptic terminal on the other side. Through this arrangement, CAMs mediate ECM and PSD signaling, influencing the dendritic spine actin network and thus the spine shape [219–225]. In turn, the spine size has a direct impact on synaptic strength, as larger spines/PSDs containing more numerous glutamate receptors have stronger effects on neuronal excitation and signal transmission [212, 226–232].

Several ECM molecules found to modulate spine formation, size, and stability through ECM receptors are also implicated in the pathology of schizophrenia (see above). CSPGs have been shown to actively stabilize dendritic spines, while their removal by enzymatic digestion results in increased spine motility [233–236]. Reelin promotes spine remodeling, impacting not only spine size and stability, but also the number of synaptic contacts per spine, effects at least in part mediated by its receptor ApoER2 [237–242]. The potential contribution of decreased Reelin expression to dendritic spine decreases in schizophrenia has long been postulated [243, 244]. Semaphorin 3A, a secreted ECM molecule expressed in PNNs, exerts a powerful effect on synapses, possibly through its plexin and neuropilin receptors [38, 197, 245–248]. Finally, and importantly, ECM proteases including tPA and MMPs have been shown to robustly affect dendritic spine stability [197]. tPA decreases spine stability, and its activation increases spine loss [249, 250]. This effect is particularly interesting as it relates to the impact of chronic stress in the amygdala and hippocampus, where tPA knockout decreases stress-induced spine loss [249, 250]. During development, MMPs play a key role in spine formation and maturation [197, 251, 252]. In mature neurons, MMPs, and their interactions with integrins, are required for spine volume changes induced by LTP and LTD [207, 253].

In summary, ECM molecules and their cell surface receptors mediate a broad range of synaptic regulatory functions impacting glutamatergic and GABAergic synapses, inhibitory neurons, and dendritic spine plasticity on excitatory neurons. The expression of several ECM molecules and their receptors involved in these functions has been shown to be altered in subjects with schizophrenia. Overall, these considerations support the hypothesis that ECM/PNN abnormalities in this disorder may disrupt synaptic functions and plasticity, perhaps leading to a dysregulation of inhibitory circuits and synaptic instability. The impact of these abnormalities is likely to be region specific, given the heterogeneous representation of these molecules in cortical and subcortical regions (e.g., [42, 55, 204–206, 254]).

## 3. ECM Pathology in Autism Spectrum Disorders

Multiple lines of evidence implicate ECM abnormalities in autism spectrum disorders, a heterogeneous group of neurodevelopmental disorders characterized by persistent deficits in social communication and social interaction and restricted, repetitive patterns of behavior, interests, or activities. Synaptic pathology is a well-established core pathological component of these disorders (e.g., [16]). Genetic studies have identified several ECM and related molecules as potential contributors to the etiology of autism. Analysis of six genome-wide association studies (GWAS) on autism implicates a number of ECM and PNN regulating molecules, including the ECM remodeling enzymes ADAMTS3, ADAMTS5, ADAMTS14, ECM molecules RELN, SEM3A, SEM4D, the hyaluronan surface receptor CD44, and OTX2, a transcription factor involved in PNN formation [255–263].

By far the strongest evidence for ECM involvement in the pathophysiology of autism comes from investigations on Reelin. GWAS, several association studies specifically investigating Reelin involvement in autism, and a meta-analysis report point to Reelin as a vulnerability gene for autism [257, 264–272]. Consistent with these findings, altered expression of Reelin and Reelin signaling pathways has been observed in the frontal, parietal, and cerebellar cortices of subjects with autism [273–275]. Reduced Reelin levels have also been shown in blood samples from subjects with this disorder [273]. Finally, the “reeler” mouse, which carries an autosomal recessive mutation in the Reelin gene, displays neurodevelopmental deficits reminiscent of psychiatric disorders including autism [273].

Emerging evidence suggests a role for heparan sulfate proteoglycans (HSPGs) in the pathophysiology of autism. Decreased HSPG expression was reported in the subventricular zone of subjects with autism [276]. Notably, this decrease was associated with increased neurogenesis in comparison to age-matched controls [276]. These findings are in agreement with an animal model of autism, the BTBR T+tf/J mouse, characterized by abnormal social behavior, communication deficits, and repetitive stereotyped behaviors as well as altered heparan sulfate expression in the subventricular zone, smaller amygdala volume, and other neurodevelopmental deficits reminiscent of those detected in autism [277–280]. Mutant mice lacking heparan sulfate show many features reflective of autism, including impaired social interaction, repetitive behavior, and deficits in ultrasonic vocalization [281]. Taken together, decreased heparan sulfate in autism may contribute to neurodevelopmental abnormalities focused on areas of cell proliferation as well as regions involved in memory and emotional processing.

## 4. ECM Pathology in Fragile-X Syndrome

Fragile-X is a single gene, inherited intellectual disability with predominant autistic symptoms [282–284]. The role that MMP-9 plays in the pathophysiology of this disorder represents a compelling example of interactions between ECM molecules and synaptic pathology in psychiatric disorders. Fragile-X results from transcriptional silencing of the* Fmr1* gene, which encodes for the mRNA binding protein “Fragile-X mental retardation protein” (FMRP) (for review see [285]). FMRP controls, in an activity-dependent manner, mRNAs encoding for pre- and postsynaptic proteins, scaffolding proteins, neurotransmitter receptors, and signaling molecules [286, 287]. Decreased FMRP expression results in elevated protein synthesis at the synapse, with loss of regulation by neuronal activity, increase in neuronal excitability, and immature, abnormal spine morphology [285, 288–293]. Converging evidence indicates that the interactions between FMRP and the matrix metalloproteinase MMP-9, known to play a role in dendritic spine plasticity in an activity-dependent manner [192, 294], may be critical to these synaptic abnormalities. Recent findings show that FMRP regulates the transport and translation of MMP-9 mRNA within the synapse: decreased FMRP results in increased MMP-9 [295]. Consistently, increased levels of MMP-9 have been reported in Fragile-X syndrome subjects while pharmacologically induced MMP-9 decrease leads to some degree of clinical improvement [296]. Elevated levels of MMP-9 were reported in the amnionic fluid of subjects who went on to develop autism later in life, including a subset of individuals without Fragile-X syndrome [297]. In parallel, Fmr1 knockout mice present with delayed dendritic spine maturation, increased MMP-9, and Fragile-X associated behaviors [295, 298, 299]. Genetic or pharmacological disruption of MMP-9 expression in these mice rescues many of these abnormalities, including dendritic spine maturation and behavioral deficits [299, 300].

## 5. ECM Pathology in Rett Syndrome

Rett syndrome is a neurodevelopmental disorder characterized by stereotypical hand movements, language regression, decreased rate of brain growth, autonomic dysfunction, and seizures [301]. Deficits in dendritic spines and synaptic plasticity have been consistently reported in subjects with Rett syndrome and in animal models of this disorder [302–306]. Rett syndrome is caused by a de novo genetic mutation in the X-linked methyl-CpG-binding protein 2 gene (MeCP2) [301, 307, 308]. Increased mRNA for RELN was reported in MeCP2 mutant mice [309]. Furthermore, increased PNN labeling with WFA was observed in the motor cortex of subjects with Rett syndrome [310]. Notably, these changes are opposite to those observed in autism and schizophrenia, suggesting that different patterns of pathological deviations of ECM composition may result in synaptic abnormalities, such as those detected in these disorders.

## 6. ECM Pathology in Mood Disorders

Mood disorders are a category of psychiatric disorders characterized by a persistent altered emotional state; they include bipolar disorder and major depression. Involvement of ECM molecules, and presence of synaptic pathology, in these disorders has been extensively documented [15, 55, 67, 311–315]. For instance, decreased Reelin expression has been reported in the prefrontal cortex, hippocampus, and cerebellum, as well as in blood, of subjects with bipolar disorder or major depression [311, 313, 316, 317]. Our postmortem studies in bipolar disorder show marked decreased of CS-6(3B3)-immunolabeled PNNs across several nuclei in the amygdala, while more moderate decreases of aggrecan-immunolabeled PNNs were observed in the accessory basal nucleus of the amygdala [55]. Furthermore, similar to schizophrenia and autism, increased levels of MMP-9 have been reported in blood samples from subjects with major depression and young subjects with bipolar disorder in a depressed state [91, 318, 319]. GWAS in bipolar disorder have identified a genetic variant of NCAN, encoding for the CSPG neurocan, as a risk factor for this disorder [314]. Consistently, NCAN gene variants are associated with manic symptoms in human subjects [320], and NCAN knockout in mice results in manic-like behaviors [320].

Intriguingly, some of the most effective treatments for mood disorders impact ECM molecules and PNN composition. For example, chronic treatment with the selective serotonin reuptake inhibitor fluoxetine, effective in treating depression and anxiety, results in decreased numbers of WFA-labeled PNNs in the hippocampus and medial prefrontal cortex of mice, accompanied by increased immature neuronal markers and dendritic spine density on interneurons [321, 322]. Fluoxetine exposure in utero has also been shown to delay the formation of PNNs during adolescence in the amygdala and hippocampus of mice [323]. Lithium, one of the most effective treatments for bipolar disorder, contributes to CSPG digestion [324, 325]. Consistently, numbers of “glial clusters” labeled with a CS-6 specific antibody (CS56), shown to be decreased in the amygdala of subjects with bipolar disorder, showed a positive correlation with lifetime exposure to lithium, raising the possibility that chronic lithium exposure may exert therapeutic effects on CSPG sulfation patterns in bipolar disorder [55].

## 7. ECM Pathology in Alzheimer's Disease

Alzheimer's disease, an irreversible late life brain disorder that progressively disrupts memory and independent living skills, is associated with dendritic spine loss [326–328]. A key neuropathological feature in Alzheimer's disease is the formation of *β*-amyloid plaques associated with mutations in the presenilin-1 and presenilin-2 genes [329, 330]. These plaques are generated by cleavage of the amyloid precursor protein (APP) by *β*-site APP cleaving enzyme-1 (BACE1) [331]. Converging evidence points to a role for HSPGs and CSPGs in the formation of amyloid plaques. Heparan sulfates regulate cleavage of APP by BACE1, and several HSPGs, including syndecan, glypican, and agrin, can be detected within amyloid plaques [332–340]. Notably, syndecan is involved in the formation of dendritic spines [341–344]. Finally, increased expression of HSPGs has been reported in postmortem brain samples from subjects with AD [335–338, 345, 346]. CSPGs, specifically CS-4, CS-6, and nonsulfated CS, have also been reported in *β*-amyloid plaques [347]. Intriguingly, a splice variant of APP corresponds to the CSPG appican, expressed primarily by astrocytes in the brain [5, 348]. Interestingly, APP cleavage by ADAM10 results in a beneficial form, amyloid-*α*, and in turn suppresses amyloid-*β* [349]. Physiologically, ADAM10 and APP are highly concentrated at the PSD site and are involved in the regulation of synaptic plasticity [350], suggesting that disrupted levels of amyloid-*α* and amyloid-*β* in Alzheimer's disease may contribute to synaptic deficits. Similarly, MMP-9 has been reported to cleave APP through *α*-secretase activity, thus promoting the nonamyloidogenic form and functioning as a protective factor from amyloid-*β* accumulation and subsequent cognitive deficits, accompanied by increased levels of presynaptic proteins [351, 352]. Furthermore, MMP-9 has been shown to degrade extracellular amyloid-*β* in amyloid plaques, providing further protection against AD pathology [353, 354].

**Figure 4:**
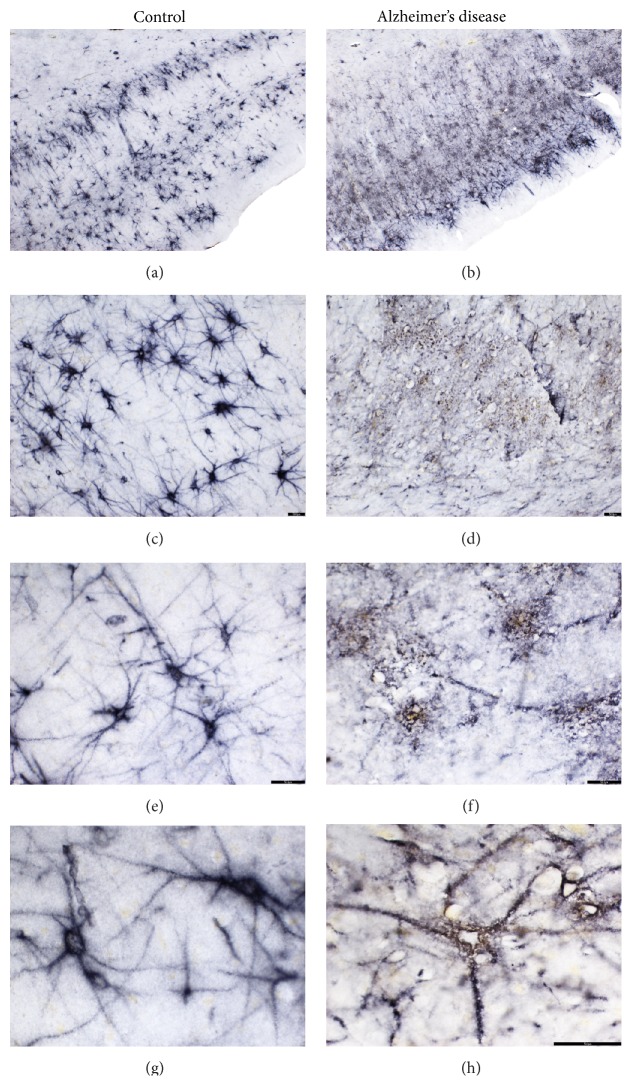
PNN structure is altered in Alzheimer's disease. Examples of WFA-labeled PNNs in the entorhinal cortex of healthy subjects (a, c, e, g) and subjects with Alzheimer's disorder (b, d, f, h). In healthy subjects, WFA-labeled PNNs are distributed across all layers of the ECx, with preferential concentration in layers II-III and layers V-VI (a). In subjects with Alzheimer's disease, WFA labeling appears to be more loosely distributed, in aggregates throughout the ECx, often suggestive of degraded PNNs (b, d, f, h).

Of relevance to this review, decreased densities of PNNs have been reported in Alzheimer's disease [355]. In particular, decreased WFA-labeled PNNs were observed in the frontal cortex of subjects with Alzheimer's disease, while densities of neurons expressing PVB were not altered [355]. These findings are in agreement with data from our group, showing dramatic decreased numbers and degraded morphology of WFA-labeled PNNs in the entorhinal cortex of Alzheimer's disease patients (Figure 4, unpublished results). Other authors have suggested that aggrecan-containing PNNs may play a protective role against tau pathology in Alzheimer's disease [356].

## 8. ECM Pathology in Epilepsy

Epilepsy encompasses a spectrum of severe to benign brain disorders characterized by disturbances of the normal pattern of neuronal activity, causing unusual emotions, behaviors, sensations, or sometimes loss of consciousness, convulsions, and muscle spasms. Compelling evidence from animal models supports the involvement of the ECM in seizure disorders. The ECM undergoes extensive remodeling in response to seizures, including increased production of CSPGs by glial cells, and cleavage of CSPGs by MMPs [357–362]. As part of such ECM remodeling, PNNs are decreased, at least in part as a consequence to aggrecan cleavage by MMPs [357, 358]. It has been proposed that ECM remodeling may allow for synaptic reorganization, such as it occurs following seizures [36, 363]. Conversely, intriguing evidence suggests that ECM abnormalities may contribute to susceptibility to seizures. For example, enzymatic PNN digestion lowers the threshold for seizure induction [358]. Similarly, inhibition of MMP activity prevents seizure induction and PNN breakdown in an amygdala kindled seizure model [363]. Kainic acid-induced seizures trigger short term CSPG changes followed by more prolonged ones, resulting in altered neurocan and phosphacan levels in limbic brain regions; this latter phase coincides with increases of spontaneous recurrent seizures [357]. In addition, mice lacking the hyaluronan synthesizing enzyme Has3 present with reduced extracellular space and display increased epileptic activity [364]. Further evidence that ECM abnormalities contribute to seizure susceptibility comes from studies on MMP-9. Levels of MMP-9 are increased in blood samples from subjects with epilepsy [365–367]. Mice lacking MMP-9 are less susceptible to induction of seizures, whereas rats overexpressing MMP-9 are more susceptible [368]. MMP-9 has been proposed as a potential therapeutic target for this disorder [365].

It is interesting to note that seizures are often comorbid with several of the disorders discussed above, including autism, schizophrenia, Fragile-X, and Rett syndrome, each presenting with ECM abnormalities. Particularly frequent in these disorders are altered levels of MMP-9 and PNN numbers [369–378]. Given the compelling relationship between ECM molecules and seizures, such comorbidity may not be surprising and, on a speculative level, may point to partially shared mechanisms.

## 9. Conclusions

The role of PNNs, and more in general ECM, represents an emerging field in the pathophysiology of psychiatric disorders. Evolving in parallel with a growing understanding of the role of the ECM in the regulation of synaptic plasticity, this field is beginning to integrate the concept of the quadripartite synapse in hypotheses on the pathophysiology of synaptic dysregulation in these disorders. Overlapping patterns of ECM abnormalities, in disorders that also share clinical features and synaptic deficits, may underlie common “end-point” mechanisms; that is, anomalies affecting one or more elements of functionally similar molecular families in each of these disorders may lead to convergent effects on synaptic functions and, potentially, clinical domains. Cell and regional specificity may be determined by nonoverlapping pathological aspects in each disorder, as well as by neurodevelopmental determinants specifying the age range at which these anomalies become pathologically relevant.
